# A community-based motivational personalised lifestyle intervention to reduce BMI in obese adolescents: results from the Healthy Eating and Lifestyle Programme (HELP) randomised controlled trial

**DOI:** 10.1136/archdischild-2016-311586

**Published:** 2017-07-07

**Authors:** Deborah Christie, Lee Duncan Hudson, Sanjay Kinra, Ian Chi Kei Wong, Irwin Nazareth, Tim J Cole, Ulla Sovio, John Gregson, Anthony S Kessel, Anne Mathiot, Stephen Morris, Monica Panca, Silvia Costa, Rebecca Holt, Russell M Viner

**Affiliations:** 1 University College London Hospitals and UCL Institute of Epidemiology and Health Care, London, UK; 2 Great Ormond Street Hospital for Children, London, UK; 3 Department of Non-communicable Disease Epidemiology, London School of Hygiene and Tropical Medicine, London, UK; 4 University College London School of Pharmacy, London, UK; 5 Primary Care and Population Health, UCL Institute of Epidemiology & Health, London, UK; 6 Population, Policy and Practice Programme, UCL Institute of Child Health, London, UK; 7 Department of Obstetrics and Gynaecology, University of Cambridge, Cambridge, UK; 8 London School of Hygiene and Tropical Medicine, London, UK; 9 UCL Institute of Epidemiology & Health, London, UK

**Keywords:** adolescent health, obesity, lifestyle intervention, child psychology

## Abstract

**Objective:**

Approximately 7% of children and young people aged 5–15 years in the UK have obesity at a level likely to be associated with comorbidities. The majority of multicomponent lifestyle programmes have limited applicability and generalisability for British adolescents.

The Healthy Eating and Lifestyle Programme (HELP) was a specific adolescent-focused intervention, designed for obese 12 to 18-year-olds seeking help to manage their weight. Participants were randomised to the 12-session HELP intervention or standard care. The primary outcome was difference in mean body mass index (BMI) (kg/m^2^) between groups at week 26 adjusted for baseline BMI, age and sex.

**Subjects:**

174 subjects were randomised (87 in each arm), of whom 145 (83%) provided primary outcome data at week 26.

**Results:**

At week 26 there were no significant effects of the intervention on BMI (mean change in BMI 0.18 kg/m^2^ for the intervention arm, 0.25 kg/m^2^ for the control arm; adjusted difference between groups: −0.11 kg/m^2^ (95% CI −0.62 to 0.40), p=0.7). At weeks 26 and 52 there were no significant differences between groups in any secondary outcomes.

**Conclusion:**

At minimum this study reinforces the need for higher level, structured interventions to tackle the growing public health burden of obesity in the UK and internationally.

The HELP intervention was no more effective than a single educational session for reducing BMI in a community sample of obese adolescents.

Further work is needed to understand how weight management programmes can be delivered effectively to young people from diverse and deprived backgrounds in which childhood obesity is common. The study has significant implications in terms of informing public health interventions to tackle childhood obesity.

**Trial registration number:**

ISRCTN: ISRCTN99840111.

**What is already known on this topic?**

- Approximately 7% of children and young people in the UK have obesity at a level likely to be associated with comorbidities.^1^
- The majority of multicomponent lifestyle programmes have limited applicability and generalisability for British adolescents.

**What this study adds?**

- The evidence-based Healthy Eating and Lifestyle intervention was no more effective than a single educational session for reducing body mass index in obese adolescents.
- Further work is needed to understand how weight management programmes can be delivered effectively to young people from diverse and deprived backgrounds in which childhood obesity is common.
- This study reinforces the need for higher level, structured interventions to tackle the growing public health burden of obesity in the UK and internationally.

## Background

Approximately 7% of children and young people in the UK have obesity at a level likely to be associated with comorbidities.^1^ Over 1% of adolescents have extreme obesity, with a body mass index (BMI) more than 3 SD above the mean.^2^ Multicomponent lifestyle modification programmes recommended by the National Institute for Health and Care Excellence^3^ are aimed at primary school children, inappropriate for adolescents developing diet and activity patterns that will stay with them into adult life. Body image and self-esteem issues become important, and behaviour change increasingly depends on individual motivation. Adolescent studies in non-UK academic/tertiary care centres with highly specialised staff involving white, middle-class, motivated families^4 5^ have limited applicability and generalisability for British adolescents. Reviews conclude a need for adequately powered high-quality trials in representative populations with integral process evaluation and appropriate lifestyle tools.^4 5^

The Healthy Eating and Lifestyle Programme (HELP) is an evidence-based multicomponent intervention focusing on enhancing motivation to change, developing self-efficacy and self-esteem for obese 12 to 18-year-olds seeking help to manage their weight. Unpublished pilot data in a clinical setting for 20 subjects aged 13–17 years showed a mean BMI reduction of 1.7 kg/m^2^ over 6 months, equivalent to a 0.4 SD effect size. We report a randomised controlled trial of HELP in a community sample of obese adolescents. Our primary aim was to assess whether HELP delivered by graduate mental health workers in the community was more effective in reducing BMI in obese adolescents than enhanced standard care. Secondary aims were to assess cost-effectiveness and impact on cardiometabolic risk and psychological function.

## Methods

### Design

We undertook a randomised efficacy trial consistent with a phase III trial in the Medical Research Council (MRC) guidance on complex interventions.^6^ Subjects were randomised to receive either HELP or enhanced care for 6 months. Details are available in the published protocol.^7^

### Intervention

HELP is a 12-session family-based weight-management programme for adolescents. Motivational interviewing^8^ and solution-focused approaches^9^ were used to increase engagement and concordance. The programme was delivered in local community settings by psychology graduates who completed a 5-day training programme on obesity and good clinical practice, with 2 of the 5 days focusing on behaviour change techniques

Session content included:

1. ‘Where and how we eat’—modifying eating behaviours/encouraging regular eating patterns;
2. ‘What we do’—decreasing sedentary behaviour/increasing lifestyle and programme activity;
3. ‘What we eat’—reducing intake of energy-dense foods/increasing healthy nutritional choices;
4. ‘Why we eat’—addressing emotional eating triggers.

### Comparator

Enhanced standard care was a 40–60 min standardised educational session incorporating Department of Health guidance on eating behaviours, healthy eating and activity, delivered by a primary care nurse (or trained nurse practitioner) in the participant’s general practice within 3 months of recruitment.

### Sample and recruitment

Eligibility was initially defined as young people (13–17 years) with BMI >98th centile for age and sex according to the UK 1990 growth reference, recruited from primary care and community settings within the Greater London area. Exclusion criteria were:

1. Diagnosed significant mental health problems/undergoing mental health treatment.
2. Chronic illness (apart from asthma unless severe requiring excessive doses of regular steroids), known secondary obesity, monogenic obesity syndrome or use of medications known to promote obesity; and those with BMI ≥40 kg/m^2^.
3. Significant learning disability or lack of command of English sufficient to render them unable to participate effectively in the intervention.
4. Participation in formal behavioural weight-management programmes in the past 12 months.

Due to slow recruitment, we widened the age range to 12–19 years and used a revised definition of obesity, that is, BMI >95th centile for age and sex and BMI <45 kg/m^2^.

We recruited through:

1. general practitioner practices, via the local Primary Care Research Network, in areas with known high prevalence of obesity;
2. paediatricians, school nurses, pharmacists and dietitians;
3. advertising on social media, the study website, community media, newsletters, community youth groups, secondary schools and colleges.

Baseline assessments were undertaken at the National Institute for Health Research Great Ormond Street Hospital Clinical Research Facility (CRF). Participants were given a £20 iTunes or high street store voucher at entry and again at completion, and reimbursed for travel costs.

### Randomisation

Participants were randomised 1:1 to the two arms and balanced for sex after baseline assessment independently of the investigators by the Health Services Research Unit University of Aberdeen.

### Outcomes

Outcomes were measured at baseline, mid-treatment (week 13), end of intervention (week 26) and 6 months postintervention (week 52) (see table 1 for details on data collected at each time point). Outcomes were assessed by clinical assessment, venepuncture and psychological questionnaires. The primary outcome (BMI, calculated from measured height and weight) was assessed by trained CRF nurses blind to treatment status.

**Table 1:** Data collection schedule

	Week 0	Week 13	Week 26	Week 52
Anthropometry	X	(X)	X	X
Motivation	X		X	X
Quality of life measure	X		X	X
Blood pressure	X	(X)	X	X
Venepuncture	X		X	
Psychological function	X		X	X
Accelerometry	X		X	

#### Primary outcome

Difference in mean BMI (kg/m^2^) between groups at the end of the intervention (week 26), adjusted for baseline BMI, age and sex. Positive differences reflect an increase, negative a decrease throughout.

#### Secondary outcomes

1. Anthropometric measures:
  - BMI (week 52)
  - BMI; SD score (zBMI) weeks 0, 26 and 52. Waist circumference; weeks 0, 26 and 52
  - Non-invasive measurement of fat mass and fat mass percentage using Tanita 418 bioimpedance scales. Fat mass (kg) was calculated from the impedance value using the formula validated in obese adolescents,^10^ and fat mass percentage was calculated dividing fat mass by body mass.
2. Health-related quality of life (HR-QOL): Impact of Weight on Quality of Life-Kids,^11^ a 27-item instrument consisting of four scales: physical comfort, body esteem, social life and family relations.^11^ Young people and parents completed separate questionnaires.
3. Psychological factors:
  - Eating Attitudes Test.^12^ Provides a total score and subscales: (1) dieting; (2) bulimia and food preoccupation; (3) oral control
  - Rosenberg Self-Esteem Scale^13^
  - Psychological health: Development and Well-being Assessment (DAWBA) online interview, which generates likely psychiatric diagnosis at the >50% likely level.^14^ Data were missing at follow-up in >50% of cases, therefore we were unable to assess the effect of the intervention on DAWBA outcomes.
4. Physical activity; Actigraph accelerometer 7-day measurement.
5. Cardiometabolic risk factors; clinical examination and venepuncture after an 8-hour fast:
  - Fasting insulin (mU/L) and glucose (mmol/L)
  - Fasting lipids (mmol/L): high-density lipoprotein cholesterol, low-density lipoprotein cholesterol and triglycerides
  - Peripheral, seated blood pressure measured using an electronic Dinamap after 20 min rest.
6. Health economic outcomes; preference-based HRQOL, resource utilisation and costs to inform a cost-effectiveness analysis (reported elsewhere).

Demographic and clinical data:

- Socioeconomic status assessed using the 2010 Index of Multiple Deprivation (IMD) for postcode.^15^
- Ethnicity, medical history, pubertal status (self-report) and weight management history.

### Data collection

Outcome data were collected at the CRF. Where subjects were unable or unwilling to attend, a home assessment was offered using portable instruments (Seca 875 Flat scales for mobile use and Seca Leicester Height Measure) to prioritise primary outcome data collection (BMI). Serious adverse events (SAE) were monitored by the research team and reported to the steering committee.

### Power and sample size

Pilot data identified a likely effect size of 1.7 kg/m^2^ reduction in BMI. Lifestyle modification programmes in obese children result in improvements in HR-QOL of 0.3–0.5 SD.^16 17^ We calculated a sample size of 126 subjects would detect a reduction of approximately 1 kg/m^2^ in BMI (equivalent to 0.5 SD) with 80% power at 5% significance. We initially inflated the sample size to account for clustering due to persistent therapist effects,^18^ assuming a therapist cluster size of 10 in both arms and an intra class coefficient (ICC)  of 0.025, inflating the size to 126*(1 + (10 − 1)*0.025)=155. To allow for 20% dropout, we initially aimed to recruit 200 subjects. The required sample size was recalculated mid-trial as mean therapist cluster size was less than 4 in the intervention arm and all control participants bar 1 had a different nurse provider. Using conservative cluster sizes of 4.5 for the intervention arm and 2 for the control arm and a within-study dropout rate of 14%, we planned to recruit at least 155 subjects in order to retain 80% power at a 5% significance threshold.

### Fidelity and compliance

Compliance was predefined as attendance at the introductory session plus five or more sessions. A qualified clinical psychologist observed each provider deliver session 1 with their first client then rated the remaining sessions from an audio recording. A Fidelity Adherence Scale based on the Motivational Interviewing Supervision and Training Scale^19^ assessed fidelity to content delivery and psychological model. Each session was self-rated by the observer from 1 (poor) to 3 (high).

### Statistical analysis

Analyses were by intention to treat. For the primary outcome, a linear regression model adjusted for age, sex and baseline BMI compared BMI at 26 weeks between groups. We fitted a random intercept at therapist level to allow for persistent therapist effects. To adjust for biases caused by missing data, our primary analysis used multiple imputation with chained equations to impute missing BMI measurements at 6 or 12 months, assuming data were missing at random. We used an imputation model with 20 imputations containing age, sex, baseline BMI, treatment group and number of attempts to contact the participant. Secondary analyses included only participants with available outcome data at baseline and 6 or 12 months.

To estimate difference in mean outcomes among compliers, we used a complier average causal effect model.^20^ This inflates intention-to-treat analyses estimates to adjust for non-compliance, avoiding problems caused by patient selection in per-protocol or on-treatment analyses. Secondary outcomes were analysed using similar methodology. Where outcomes were binary or ordinal, we used logistic or ordinal logistic regression models. For highly skewed continuous variables we used robust SEs. All analyses were performed using Stata V.13.0 (StataCorp, College Station TX).

## Results

**Figure 1:**
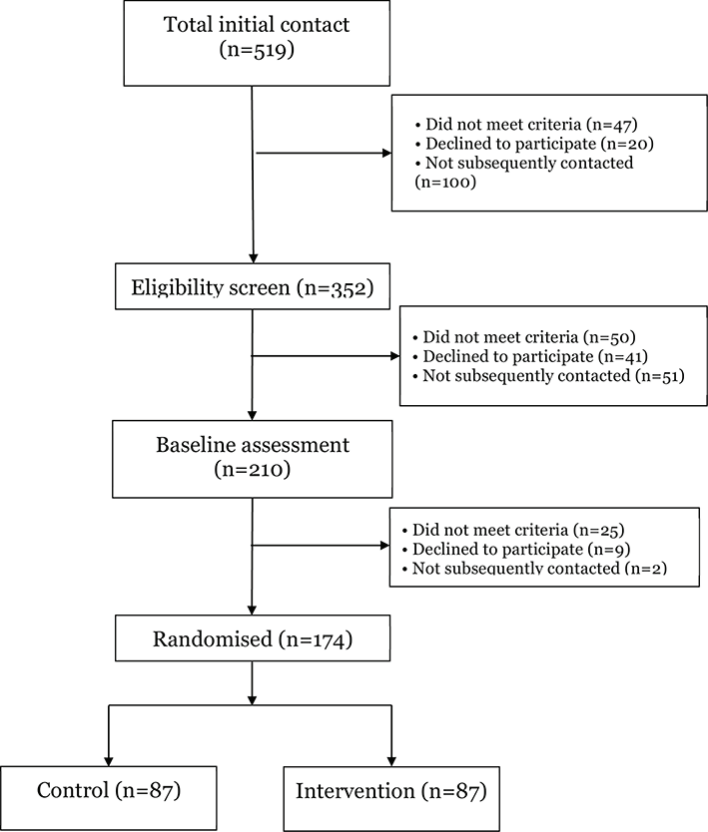
CONSORT (Consolidated Standards of Reporting Trials) Recruitment flow diagram.

Five hundred and nineteen young people or families contacted the team. Three hundred and fifty-two completed a telephone screening interview. Two hundred and ten were invited to attend a baseline assessment (figure 1). A small number of young people failed to provide accurate contact details after their initial indication of interest. They were therefore not subsequently contacted.

**Figure 2:**
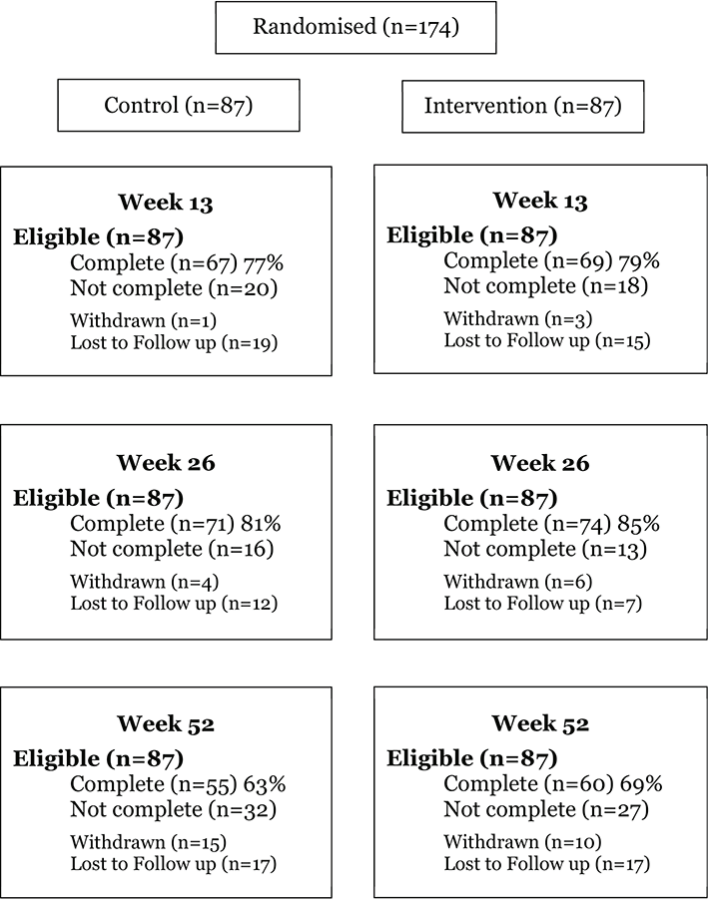
CONSORT (Consolidated Standards of Reporting Trials) flow chart of follow-up of the 174 participants randomised in the Healthy Eating and Lifestyle Programme study.

One hundred and seventy-four young people (88%) were randomised into the trial with 87 in each arm. Primary outcome data on BMI at 26 weeks were collected on 145 (83%) participants (figure 2). Mean age at baseline was 15 years. Mean BMI was 32.0; 63% of participants were female (table 2). The two arms were well balanced with respect to all baseline characteristics.

**Table 2:** Baseline characteristics of participants by treatment group

	Control (n=87)	Intervention (n=87)
	Median (IQR) or n (%)	Median (IQR) or n (%)
Age (years)	15 (14–17)	15 (13–17)
Female	55 (63%)	54 (62%)
Ethnicity		
Black	34 (41%)	18 (22%)
Asian	13 (16%)	21 (25%)
White or mixed	36 (43%)	44 (53%)
Unknown	4 (5%)	4 (5%)
Anthropometry		
BMI (kg/m^2^)	32.0 (29.2–34.6)	32.0 (28.7–35.5)
BMI z-score	2.8 (2.4–3.3)	2.8 (2.5–3.3)
Weight (kg)	88.9 (80.0–100.4)	84.4 (76.8–100.9)
Waist circumference (cm)	99.0 (92.1–107.0)	98.0 (90.6–108.7)
Estimated fat percentage	44.1 (37.4–47.5)	42.8 (39.0–49.0)
Psychological scales		
EAT-26		
Eating attitude score (0–39) (n=173)	11 (6–18)	10 (6–17)
Dieting scale score (0–39) (n=173)	7 (3–11)	6.5 (3–12)
IWQOL-Kids		
Participant-reported global score (0–100) (n=171)	74 (62–84)	77 (68–87)
Parent-reported global score (0–100) (n=156)	74 (62–86)	70 (56–81)
Rosenberg global score (0–30) (n=165)	18 (15–20)	18 (15–23)
Cardiometabolic risk factors		
Blood glucose (mmol/L) (n=173)	4.4 (4.2–4.6)	4.5 (4.3–4.9)
Insulin (mmol/L)	11.7 (8.2–18.9)	14.2 (7.8–20.4)
LDL-C (mmol/L) (n=171)	2.5 (2.1–3.3)	2.7 (2.2–3.1)
HDL-C (mmol/L) (n=171)	1.1 (1.0–1.3)	1.1 (1.0–1.3)
Triglycerides (mmol/L) (n=173)	1.0 (0.8–1.2)	1.0 (0.7–1.4)

All n’s are 174 except where stated.

Rosenberg scale—higher score means higher self.

BMI, body mass index; EAT-26, 26-Item Eating Attitudes Test; HDL-C, high-density lipoprotein cholesterol; IWQOL-Kids, Impact of Weight on Quality of Life-Kids; LDL-C, low-density lipoprotein cholesterol.

On average, participants attended 10 of the 12 HELP sessions, with 27 young people (31%) attending all 12 sessions and 69 (79%) meeting the criteria for compliance. Seventy-six per cent of observed sessions were rated as good.

The effect of the intervention on BMI at 26 weeks adjusted for baseline age, sex and BMI was −0.11 kg/m^2^ (95% CI −0.62 to 0.40, p=0.7), indicating the effect of the intervention was non-significant (table 3). Positive changes reflect an increase, negative is a decrease throughout. Twenty-nine 6-month BMI measurements were imputed. Throughout the results from complete case analyses mirror almost exactly those using multiple imputations.

**Table 3:** Estimated effect of intervention on primary and secondary outcomes (positive is an increase, negative is a decrease)

Variable	Number with both baseline and outcome data control/intervention	Mean change in control group	Mean change in intervention group	Adjusted difference between intervention and control (95% CI)*	p Value
Primary outcome					
BMI at 26 weeks (kg/m^2^)	71/74	0.25	0.18	−0.11 (−0.62 to 0.40)	0.7
Secondary outcomes (at 26 weeks unless specified)					
BMI at 52 weeks (kg/m^2^)	55/60	0.80	0.50	−0.22 (−1.05 to 0.61)	0.6
BMI z-score	71/74	−0.03	−0.02	0.00 (−0.07 to 0.07)	>0.9
Waist circumference (cm)	61/64	0.16	−0.11	−0.72 (−3.28 to 1.84)	0.6
Weight (kg)	71/74	1.77	2.00	0.07 (−1.51 to 1.65)	0.9
Fat percentage	55/60	1.25	0.71	−0.21 (−1.57 to 1.14)	0.8
Supine systolic BP (mm Hg)	68/73	2.19	3.04	0.01 (−3.24 to 3.26)	>0.9
Supine diastolic BP (mm Hg)	68/73	3.41	2.08	−1.21 (−4.63 to 2.20)	0.5
Eat-26 (0–39)					
Eating attitude score	65/69	−0.72	−0.84	0.15 (−1.24 to 1.54)	0.8
Dieting scale score	65/69	−1.03	−1.26	−0.09 (−2.32 to 2.14)	0.9
IWQOL-Kids (0–100)					
Participant-reported global score	63/69	6.22	5.94	0.14 (−3.53 to 3.81)	0.9
Parent-reported global score	52/50	4.37	7.46	0.51 (−3.91 to 4.93)	0.8
Rosenberg global score	61/64	1.77	1.45	−0.25 (−1.70 to 1.20)	0.7
Insulin (IU/L)	56/61	4.31	3.90	1.51 (−1.46 to 4.48)	0.3
LDL cholesterol (mmol/L)	53/61	−0.20	−0.13	0.09 (−0.07 to 0.26)	0.3
HDL cholesterol (mmol/L)	53/61	−0.08	−0.04	0.02 (−0.06 to 0.09)	0.7
Triglycerides (mmol/L)	55/61	−0.01	−0.07	0.08 (−0.08 to 0.24)	0.3

IWQOL-Kids—Higher score means higher weight-related quality of life.

BMI, body mass index; BP, blood pressure; EAT-26, 26-Item Eating Attitudes Test; HDL, high-density lipoprotein; IWQOL-Kids, Impact of Weight on Quality of Life-Kids; LDL, low-density lipoprotein.

*95% confidence interval.

**Figure 3:**
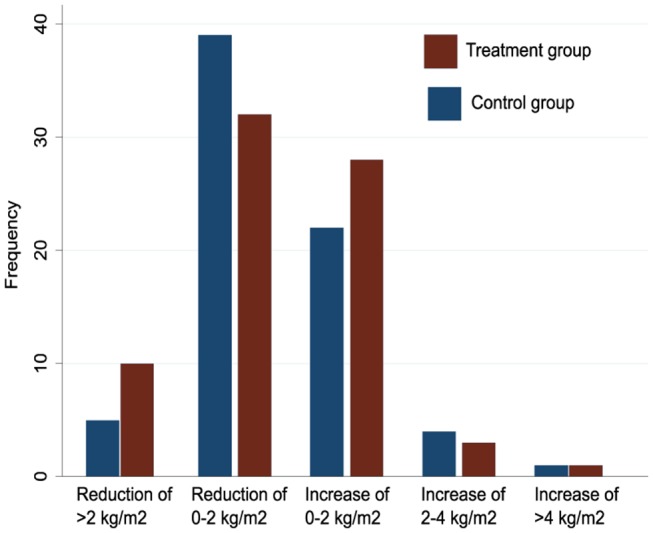
Distribution of changes in body mass index from baseline to 26 weeks in control and treatment groups among 145 participants with measurements at both time points.

The effect among compliers was −0.14 kg/m^2^ (95% CI −0.78 to 0.51, p=0.7) and the distributions of BMI change at 26 weeks in both arms were very similar (figure 3). Among 145 patients with BMI measurements at baseline and 6 months, the measurement at 6 months was lower in 85 (59%) patients. When we compared the number of patients in various categories of change in BMI (>2 kg/m^2^ reduction, 0–2 kg/m^2^ reduction, 0–2 kg/m^2^ increase, 2–4 kg/m^2^ increase, >4 kg/m^2^ increase), there was no evidence of a difference in the distribution of patients among the various categories.

### Secondary outcome analyses

There were no significant differences between arms for BMI at 52 weeks (effect −0.22 kg/m^2^, 95% CI −1.05 to 0.61), weight (−0.72 kg, 95% CI −3.28 to 1.84), waist circumference (0.07 cm, 95% CI −1.51 to 1.65) and fat percentage (−0.21%, 95% CI −1.57 to 1.14) (table 3). There were no significant differences for blood pressure, psychological function and measures of lipid and glucose metabolism. There were no SAEs reported related to participation in the study.

## Discussion

The intervention, previously shown to be promising in clinical practice, did not reduce BMI or improve psychological or cardiometabolic function. Attrition was low compared with other paediatric obesity studies.^21^

We recalculated the estimated dropout rate at the time of recalculation of the sample size. The ICC estimates of 0.025 and a mean cluster size of 10 was a conservative estimate to ensure that we had enough statistical power; at the end of the study, our estimated ICC was <0.01, and our maximum cluster size was 7 suggesting that we had greater than anticipated statistical power.

We were able to exclude clinically important effect sizes for the intervention, such as differences in BMI of 0.6 kg/m^2^ at 6 months or 1 kg/m^2^ at 12 months.

Fidelity of delivery was good with approximately 80% of participants receiving the intended ‘dose’ of intervention.

Our study was subject to a number of limitations. We widened our eligibility criteria to include adolescents with morbid obesity; however, only 9 participants (4.6%) had baseline BMI between 40 and 45 kg/m^2^. Attrition at 52 weeks reduced the power to identify an effect of the intervention, although it is likely that direction of bias from attrition would have been to preferentially retain those in whom the intervention had been effective and inflate any identified effect. While we had full data on the primary outcome follow-up, we were unable to collect sufficient data on physical activity and psychological morbidity. Given the absence of significant effects on other secondary outcomes, it is unlikely that the intervention would have had an effect on physical activity and psychological morbidity.

### Comparison with the literature

Programmes that include adolescents have more variable outcomes than those for earlier childhood.^4 5 22^ Our findings are similar to a number of other published weight management trials of a lower quality. A recent systematic review reported 28 of 31 studies had unclear or high risk of bias due to lack of information/procedures to ensure adequate randomisation/blinding of outcome assessments.^22^ Recent meta-analyses suggest lifestyle modification programmes in children are associated with a BMI reduction from 1.15 kg/m^2^ 23 to 1.3kg/m^2^.^5^ Systematic reviews suggest lifestyle modification interventions improve quality of life^23^ and may have small effects on lipids and insulin.^5^ In contrast, we found no impact on BMI, lipids or quality of life. There were small non-significant increases in self-esteem. Other studies have shown a benefit in self-esteem measures often in the face of little change in obesity measures.^24^

Possible explanations for null findings may relate to (A) intervention, (B) methodology or (C) population. It is possible the trial did not provide an adequate test of the intervention; however, it was adequately powered and methodologically robust. Due to slow recruitment, the age range was widened and this, together with other dropouts, may have impacted upon the representativeness of the sample and generalisability of results. It is likely that those who completed were more motivated in both arms, and any bias would be against HELP as that required more input.

A large proportion of control participants achieved a small BMI reduction raising the possibility that the comparator had some effect.

It is possible the population was different from interventions that have identified an effect. Our sample was highly deprived, with approximately 40% in the most deprived IMD quintile (20% expected) and around half from black or minority ethnic groups. Mental health problems were identified in 34% at baseline, despite excluding previously known mental health conditions. This is considerably higher than the general population.^23^ Most published studies have been undertaken in hospital or academic settings^5^ in the USA dominated by more affluent white families.^4^ Childhood obesity interventions based on education and personal behavioural change are less likely to be effective in more deprived populations^25^ as poorer families may face structural and cultural barriers to lifestyle change including financial barriers and fewer family/community resources.

Finally, results may be explained by delivery, content and intensity factors. Our pilot data were obtained from a small clinical programme delivered by highly trained clinical psychologists. Less well-qualified graduates only had 2 days training in behaviour change techniques (and the intervention manual) with regular supervision; however, reduction in therapist skill level may have contributed to our null results.

## Conclusions

HELP was no more efficacious reducing BMI in a community sample of obese adolescents than a single educational session adding to the existing evidence base of lack of effectiveness of weight management trials for children and adolescents. Further work is needed to understand how weight management programmes can be delivered effectively to young people from diverse and deprived backgrounds in which childhood obesity is common. At minimum, this study reinforces the need for higher level, structured interventions to tackle the growing public health burden of obesity in the UK and internationally.
